# Supplementary material for: Topological control of extreme waves
Source: Nat Commun. 2019 Nov 8;10:5090. doi: 10.1038/s41467-019-12815-0 (PMC6841676; doi:10.1038/s41467-019-12815-0)
Supplement: Supplementary file 1 — Supplementary Information [file 41467_2019_12815_MOESM1_ESM.pdf]

Topological Control of Extreme Waves - Supplementary Information

Giulia Marcucci,[1,2,*] Davide Pierangeli,[1,2] Aharon J. Agranat,[3]
Ray-Kuang Lee,[4] Eugenio DelRe,[1,2] and Claudio Conti[1,2]

[1] *Department of Physics, University Sapienza, Piazzale Aldo Moro 5, 00185 Rome, Italy*
[2] *Institute for Complex Systems, Via dei Taurini 19, 00185 Rome, Italy*
[3] *Applied Physics Department, Hebrew University of Jerusalem, 91904 Israel*
[4] *Institute of Photonics Technologies, National Tsing Hua University, Hsinchu 300, Taiwan*

(Dated: October 24, 2019)

Potentially Competing Effects: Modulation Instability and Losses

We perform experiments - and validate them by numerical simulations - to prove that our results are genuinely caused by a NLSE box evolution, and they are not due to modulation instability (MI) arising from noise in the central part of the box. Supplementary Figure 1 reports the outcomes. MI generates transversal periodic waves; DSWs occur in strongly nonlinear regimes and present fast non-periodic oscillation. Suppl. Figs. 1a-d show the different behaviors of such phenomena, occurring on two distinct spatial scales in our experiments. It turns out that MI from spontaneous noise affects light propagation only for very large beam waists, much larger than the ones analyzed in the main manuscript, because the period of generated waves is comparable to the waist corresponding to Suppl. Fig. 1d. Suppl. Fig. 1e illustrates the experimental gain related to Suppl. Fig. 1a at $\bar{t} = (30 \pm 2)$s, computed through the expression [17]

$$G(k_x) = \frac{1}{L} \log \left[\frac{\hat{A}(k_x, z = L)}{\hat{A}(k_x, z = 0)} \right], \tag{1}$$

with $L = 2.5$mm the propagation distance, $\hat{A}$ the Fourier transform of the field envelope and k_x the spatial momentum. A possible waist threshold value, which separates the nonlinear and the MI dynamics, is also established by the the comparison of the spectral weights in Suppl. Fig. 1f. Spectral weights are computed as maximum absolute values of $\hat{A}$ field for the DSW and MI momenta, and they indicate a change of dominating effect around $W_0 \sim 150\mu$m. This is also proved numerically in Supplementary Figures 2a-e: above $W_0 \sim 150\mu$m [Suppl. Figs. 1a-d] MI alters significantly light propagation, while below, in Suppl. Fig. 1e [corresponding to Fig. 2a in the main manuscript, but with initial perturbative noise], we cannot appreciate any modification of nonlinear dynamics.

Another effect that could modify the nonlinear dynamics is the presence of loss, as shown in [47], where the dissipation significantly affects the local genus of the breather structure. Adding losses to Eq. (8) of the main text, we obtain

$$\imath \partial_z A + \frac{1}{2k} \nabla^2 A + \frac{k}{n_0} \delta n(I) A = -\imath \frac{\alpha}{2} A. \tag{2}$$

The KLTN photorefractive crystal is transparent above $\lambda = 380$nm, and the copper doping introduces a small absorption from $\lambda = 550$nm to $\lambda = 800$nm, where the value of the absorption coefficient α is approximately $\alpha = 2\text{cm}^{-1}$. It turns out that losses need a propagation length $L_{\text{loss}} = \alpha^{-1}$ to be effective for the pump laser ($\lambda = 633$nm), that is, one order of magnitude higher than the crystal length L, while the reference laser beam, generating the box, propagates without losses ($\lambda = 532$nm).

Rogue Waves and Soliton Gas Analysis in the Box Problem

Rogue waves are waves of unusually high intensity $|\psi_{\text{RW}}|^2$, whose probability density function (PDF) does not decay exponentially (linearly on the semilogarithmic scale used in Supplementary Figures 2f-i), but presents a tail at highest intensity values [30].

The statistical properties of the intensities illustrated in Suppl. Figs. 2f,g confirm the occurrence of RWs in the small box regime, both with initial noise [Suppl. Fig. 2f] and without [Suppl. Fig. 2g]. The latter case is widely treated in the main manuscript, where the emergence of PSs is proved, and the agreement with analytical PS profile is demonstrated both in intensity and in phase outlines. The question regarding the deterministic nature of models generating PSs, as the focusing NLSE with initial rectangular conditions, and so the fact that the PS emergence is wholly predictable and does not exhibit a statistical rarity, makes the debate on considering PSs RW prototype still open. Another conventional criterion for RWs is $|\psi_{\text{MAX}}|^2 > 8|\psi_{\text{BG}}|^2$, with $|\psi_{\text{MAX}}|^2$ the intensity peak and $|\psi_{\text{BG}}|^2$ the background intensity [40]. Figs. 4,5 prove that the PSs generated in simulations and experiments fulfill this requirement.

The same analysis can be done for the ABs generation, shown in Figs. 2,3, whose intensity PDF are reported in Suppl. Figs. 2h,i.

Last point we want to discuss is about the formation of SGs in the long-term propagation reported in Figs. 1a,2a,3c. Solitons are propagation-invariant waves with particle-like interactions. This means that, when many solitons form a disordered finite-density ensemble rather than a well-ordered modulated soliton lattice, they resemble a gas of particles. The definition of SG has required a huge effort from scientific community to be established. G. A. El *et al.* in 2005 derived the kinetic

equations for SGs in physical systems described by integrable nonlinear wave equations [36].

About the box problem and its evolution in terms of genera, the leading theoretical paper is [40], and an introductory part of the related analysis is reported in the very first paragraphs of our manuscript (main text). In [40], the authors associate the long-time asymptotic solution ψ with a "breather gas" and numerically observe the presence of higher-order RWs with maximum height $4 < |\psi_{\mathrm{MAX}}| < 5$ in the regions with $g \geq 4$. Their numerical simulations suggest that the pattern of the $\xi - \zeta$ plane (splitting into the regions of different genera) persists as ζ increases, and therefore $g \sim \zeta$ asymptotically.

In 2018, A. A. Gelash *et al.* studied a statistically homogeneous SG with essential interaction between the solitons [33]. The model used by the authors is a focusing NLSE, and they generated ensembles of $N-$soliton solutions ($N \sim 100$) by using the Zakharov-Mikhailov variant of the dressing method. Through such a mathematical description, it was demonstrated that spontaneous noise-induced MI of a plane wave generates SGs [34].

In the KdV theory, the thermodynamic type infinite-genus limit of finite-band potentials leads to the kinetic description of a SG [36]. Very recently, I. Redor *et al.* [37] showed that it is possible to produce in a laboratory a SG described in statistical terms by integrable turbulence in hydrodynamics. In optics, the focusing NLSE counterpart of this theory would include the breather gas description, yet to be developed, providing insights on NLSE turbulence, which is subject of active research [39].

We aim at improving the breather gas description in terms of topological control. Further information will be reported in future work.

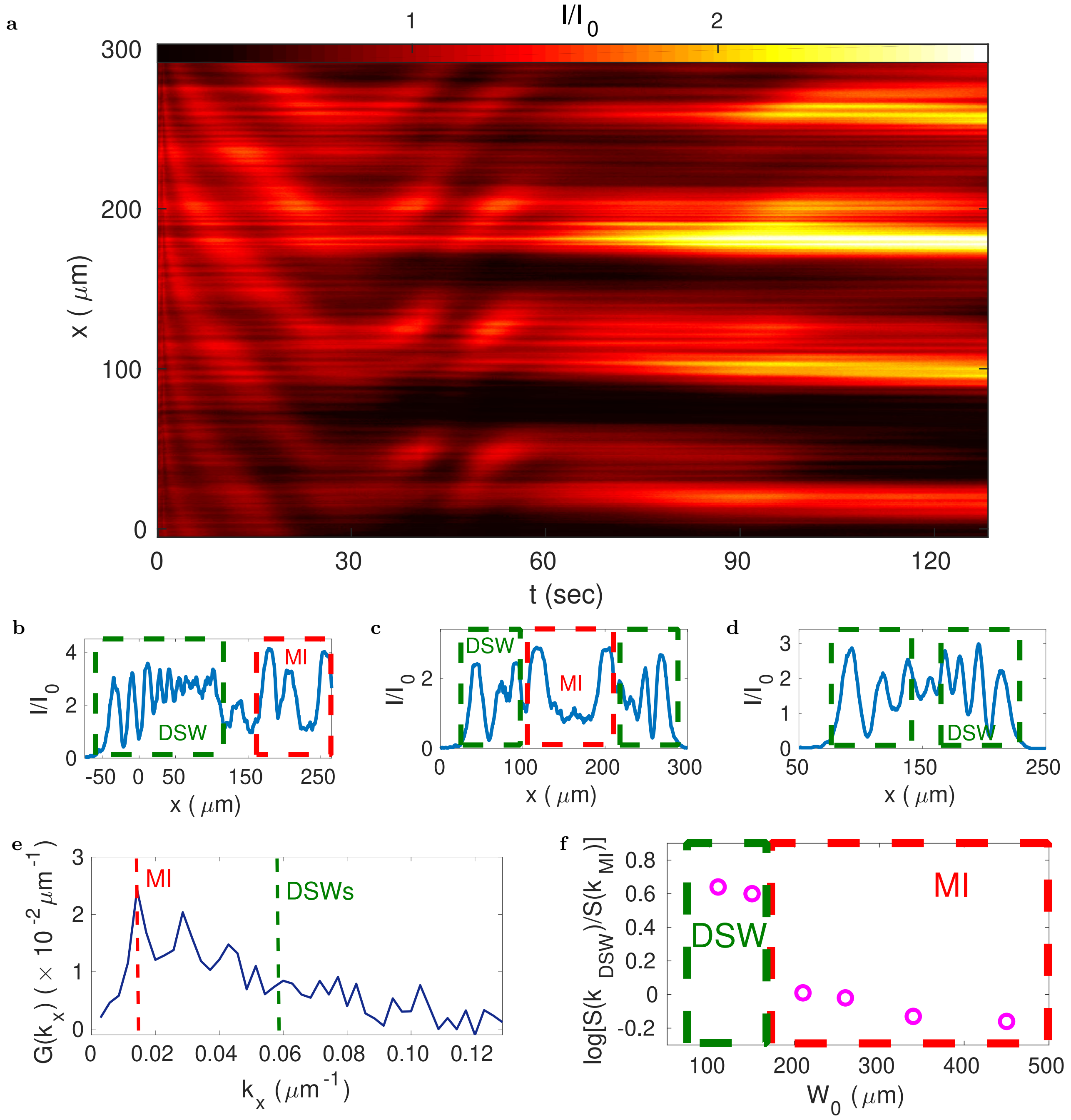

Supplementary Figure 1. **Experiments at large beam waists and modulation instability.** **a** Observation of normalized optical intensity for an initial box-shaped beam propagation in KLTN photorefractive crystal, with a waist as large as the transverse crystal length (2.1 mm). Axis t is time of output detection, x is the transverse direction. In this regime, MI dominates light propagation. **b-d** Observations of normalized optical intensity for initial box-shaped beams of waists (**b**) $W_0 = 450\mu$m, (**c**) $W_0 = 260\mu$m and (**d**) $W_0 = 150\mu$m at fixed time $\bar{t} = (30 \pm 2)$s. If MI from intrinsic noise generates periodic waves between the two DSWs in (**b**,**c**), with a period larger than the DSWs oscillation length, (**d**) represents a threshold waist value, below which MI does not affect the dynamics. **e** Experimental gain [see Eq. (1)] for the initial infinite box-shaped beam versus spatial momentum, at time $\bar{t} = (30 \pm 2)$s. **f** Logarithm of the ratio between the two spectral weights, namely, the maximum absolute values of the Fourier transform of the optical field for the DSW and MI momenta. It highlights two beam waist intervals: the one below 150μm, where MI does not affect light propagation, and the one above 150μm, where MI dominates the dynamics.

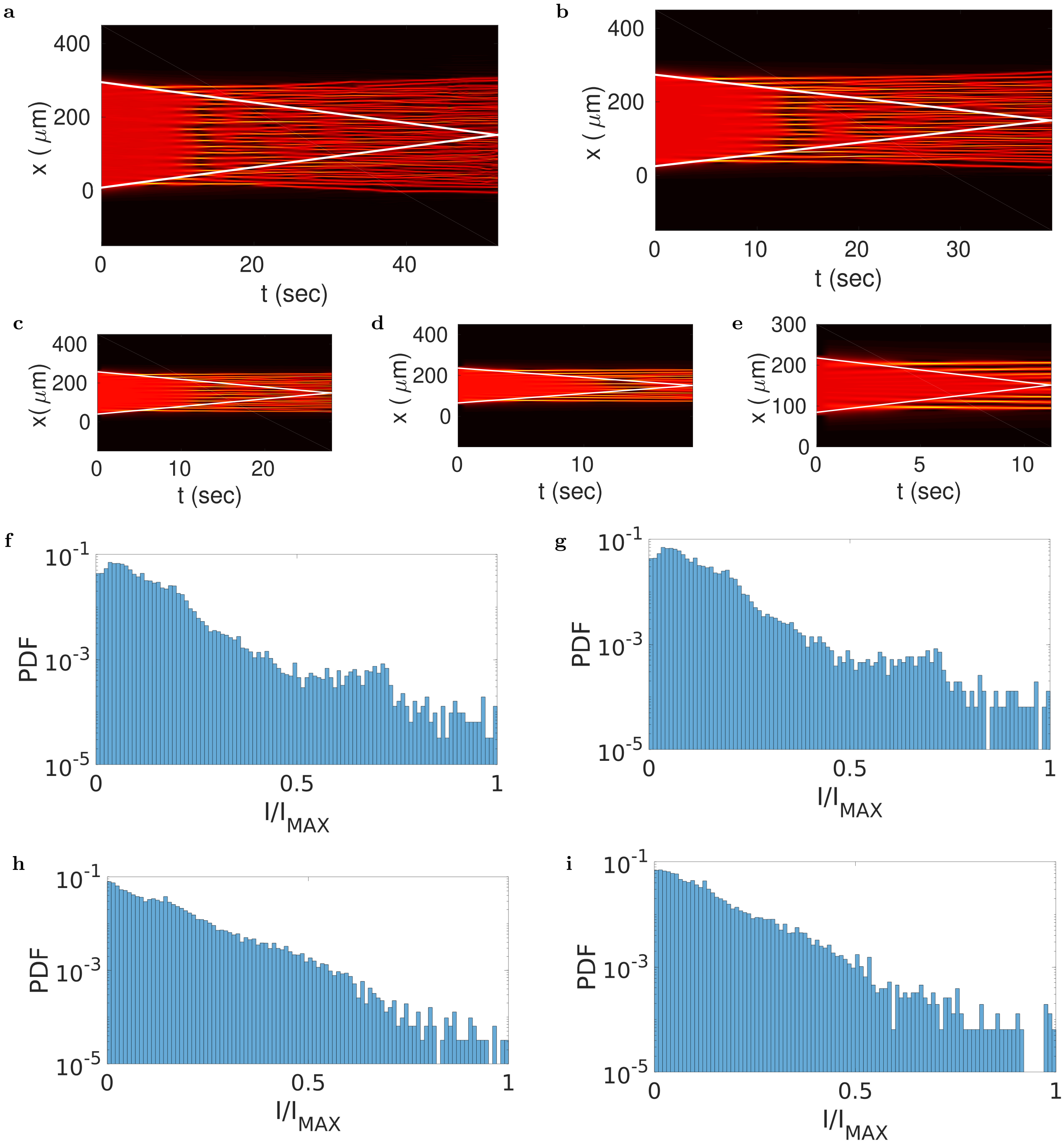

Supplementary Figure 2. **Modulation instability and rogue waves emergence.** **a-e** Numerical simulations of the final states after a propagation distance $L = 2.5$mm for beam waists (**a**) $W_0 = 300\mu$m, (**b**) $W_0 = 260\mu$m, (**c**) $W_0 = 220\mu$m, (**d**) $W_0 = 180\mu$m and (**e**) $W_0 = 140\mu$m. Axis t expresses time of detection, while x is the beam transverse coordinate. The initial conditions are box-shaped beams with small-amplitude pertubations, implemented as Gaussian random noise. White lines represent the shock separatrices. MI generates periodic waves between the two DSWs in (**a-d**), but does not affect the dynamics in (**e**). **f-i** Probability density functions of the optical intensity I/I_{MAX} over a two-dimensional spatio-temporal computational window, centered at $x = 150\mu$m, for $t \in [0\mathrm{s}, 60\mathrm{s}]$, and I_{MAX} the peak intensity (semilogarithmic scale). Initial conditions are: (**a**,**b**) $W_0 = 20\mu$m (small-waist regime) with and without random noise, respectively; (**c**,**d**) $W_0 = 100\mu$m (large-waist regime) with and without random noise, respectively. All the configurations present a significant deviation from the exponential distribution, signature of presence of RWs.
